# Conforming to AI vs. humans: The moderating effects of social exclusion and task type

**DOI:** 10.1371/journal.pone.0355478

**Published:** 2026-08-31

**Authors:** HeeJun Lee, Hyeontaek Oh, Yoonkyung Jeong

**Affiliations:** Department of Psychology, Catholic University of Korea, Seoul, South Korea; Shanghai Jiao Tong University, CHINA

## Abstract

Conformity is often understood as a socially motivated process; however, individuals increasingly conform not only to humans but also to artificial intelligence (AI). It remains unclear whether the same motivational and task-related mechanisms underlie conformity across these sources. To examine whether conformity to AI reflects task- and motivation- dependent processes similar to those underlying conformity to humans, we conducted an online conformity experiment in which we manipulated opinion source (AI vs. human) and task type (objective vs. subjective), while measuring participants’ self-reported experiences of social exclusion as a potential moderator. Participants (N = 354) were randomly assigned to either an AI or human opinion-source condition and completed a series of judgment tasks. The results showed that social exclusion reduced conformity to AI-generated opinions, but not to human opinions. In contrast, task type did not significantly moderate the effect of opinion source. These findings suggest that conformity to AI may be shaped by mechanisms that differ from those underlying traditional interpersonal conformity, particularly under conditions of social disconnection. More broadly, the findings indicate that responses to AI- generated opinions may depend not only on social-affiliative motives but also on informational or epistemic evaluations of the source.

## Introduction

Conformity has traditionally been understood as a fundamentally social process. Individuals adjust their judgments and behaviors not merely to improve accuracy, but also to maintain social alignment and secure belonging within a group [1]. In this sense, conformity functions as an affiliative mechanism that helps individuals navigate social environments and sustain interpersonal connections [2].

However, with the growing use of AI in everyday decision-making, individuals are increasingly exposed to influence from non-human agents. As AI becomes more integrated into everyday decision-making contexts, algorithm-generated recommendations may shape how people form their judgments. Emerging evidence suggests that individuals sometimes align their judgments with artificial agents in ways that resemble social conformity [3]. This invites consideration of whether conformity toward AI serves the same social function as conformity toward humans.

To address this question, it is helpful to consider broader theoretical models of conformity, as prior research on interpersonal influence suggests that susceptibility to others’ opinions is inherently multifactorial. Although Advice Response Theory was developed in the context of advice-taking, its core premise—that susceptibility to others’ input depends on the interaction of source-, message-, and receiver-related factors—offers a useful lens for understanding conformity as a form of influence response. Specifically, Advice Response Theory delineates three primary dimensions that shape opinion susceptibility [4,5]: opinion source–related factors, such as perceived credibility or the identity of the opinion provider; message-related factors, such as the perceived efficacy of the opinion in resolving the issue; and receiver-related factors, such as internal motivations. These three dimensions exert distinct influences on opinion susceptibility and may also interact with one another. Therefore, a multifactorial approach is needed to understand conformity to AI-generated opinions, with attention to opinion source characteristics, message properties, and individual-level motivations.

Within this perspective, opinion source–related factors play a central role in shaping conformity. The perceived credibility of an opinion source has been shown to increase susceptibility to social influence [6]. Whether the source is human or AI is one source characteristic that may be associated with perceived credibility. Early conformity research primarily conceptualized social influence in terms of human opinion sources [7]. However, technological developments have expanded the range of influence agents beyond humans. Artificial intelligence is increasingly perceived as a social actor capable of exerting influence. This idea aligns with the Computers Are Social Actors (CASA) paradigm, which posits that individuals respond to AI in social ways despite recognizing that these systems lack genuine intent or biological agency [8]. Consistent with this perspective, empirical studies show that individuals can conform to non-human agents, including both physical robots [9] and AI systems [3].

However, recognizing AI as a social entity does not necessarily lead to a uniform response. Prior research suggests that evaluations of AI opinions are characterized by two contrasting tendencies: algorithm aversion and algorithm appreciation. On the one hand, algorithm aversion refers to the tendency to discount AI judgments in favor of human input, particularly after observing algorithmic errors [10]. Individuals often hold algorithms to stricter performance standards than humans and exhibit greater moral resistance toward algorithmic decisions [5,11]. On the other hand, algorithm appreciation—often discussed in relation to algorithm trust— reflects a growing inclination to rely more heavily on AI-generated outputs than on human advice [12]. For example, in financial decision-making, individuals followed algorithmic advice more closely than crowdsourced advice, even when the content was identical [13]. Taken together, these divergent findings suggest that responses to AI advice may depend on task context, underscoring the importance of considering task characteristics when examining conformity to AI versus human opinions.

One possible explanation for these divergent findings is that people perceive AI and humans as possessing domain-specific expertise. Individuals tend to show greater conformity toward AI in objective and measurable tasks, whereas human opinions are often preferred in subjective and ambiguous domains [14,15]. These patterns suggest a perceived specialization between AI and humans, whereby AI is viewed as more competent in analytical contexts and humans as better suited for subjective judgments. Importantly, these task-dependent patterns further suggest that credibility is not a fixed attribute of the source but is evaluated relative to domain-specific expectations.

Conformity has often been conceptualized as reflecting social motivation. Individuals conform not only because others may be correct, but also because alignment fosters social connection and reduces the risk of exclusion. Experiences of social exclusion heighten sensitivity to social cues and increase behaviors that facilitate reconnection, such as mimicry, cooperation, and conformity [16–18].

However, social exclusion does not uniformly elicit reconnection motives. According to the Multi-Motive Model, exclusion can activate either reconnection or self-protective responses depending on situational appraisal [19]. When individuals perceive opportunities for meaningful reconnection, they tend to engage in affiliative behaviors. In contrast, when reconnection is not perceived as attainable, exclusion can trigger defensive responses such as aggression or withdrawal [20,21].

Critically, this framework suggests that conformity following social exclusion may depend on whether the influence source is perceived as an attainable target for social reconnection. Although AI can sometimes be treated as a social actor, it may not be perceived as capable of providing meaningful social acceptance or relational reciprocity. Thus, while conformity traditionally serves an affiliative function [17], it remains unclear whether socially excluded individuals would similarly conform to AI advice.

Taken together, these perspectives suggest that social exclusion may differentially shape conformity depending on whether the influence source is perceived as affording meaningful social reconnection. However, existing research has primarily examined responses to AI in relation to task performance and domain competence, leaving unresolved how individuals’ social motivational states influence their responsiveness to artificial versus human opinions. This question is theoretically consequential because conformity has often been conceptualized as reflecting affiliative motivations grounded in social needs. Building on this perspective, the present study investigates how conformity to AI and human opinions is moderated by two distinct factors: task type and social exclusion. Specifically, we test whether task type (objective vs. subjective) and individuals’ experiences of social exclusion differentially alter their responsiveness to human versus artificial agents. This approach allows us to examine whether alignment with AI reflects relatively greater cognitive– informational considerations related to task performance, or whether it is similarly shaped by social–affiliative motivations that typically underpin conformity to human opinions, particularly under conditions of social exclusion. Based on prior research suggesting greater reliance on AI opinions in objective task contexts, we expected conformity toward AI opinions to be greater in relatively objective than subjective tasks. In addition, drawing on the Multi-Motive Model, we examined whether social exclusion would differentially relate to conformity depending on whether opinions originated from AI or human sources. Specifically, because conformity has traditionally been understood as serving affiliative functions, social exclusion was expected to show a stronger association with conformity toward human opinions than toward AI opinions.

## Method

### Participants and design

A total of 354 adults residing in the Republic of Korea participated in an online experiment (115 men, 239 women; M = 30.54, SD = 8.34, range = 19–68). Participants were randomly assigned to either the AI opinion group (N = 167) or human opinion group (N = 187) for the experiment. All procedures complied with the ethical standards of the Declaration of Helsinki. This study was approved by the Institutional Review Board of the Catholic University of Korea (1040395-202510-05). Participants were recruited between November 17, 2025, and January 30, 2026. All participants provided informed consent prior to participation.

### Material

*Conformity.* Conformity was quantified using a normalized distance-reduction index reflecting the degree to which participants adjusted their judgments toward the reference value. Specifically, conformity was computed as follows:


$$\begin{array}{c} Conformity=\frac{\left|Pre-M|-|Post-M\right|}{\left|Pre-M|+|Post-M\right|} \end{array}$$

where *Pre* and *Post* denote participants’ judgments before and after receiving the opinions, respectively, and M represents the mean value of the four presented opinions within each trial. The four opinions presented within each trial were designed to remain close in value to minimize within-trial variability. Accordingly, the mean value of the four opinions was used as the reference value (*M*) in the conformity calculation. Because the presented opinion values were decimal values whereas participants’ responses were made on integer Likert-type scales, the denominator of the conformity index could not equal zero by design.

*Social Exclusion Experience Scale.* To measure participants’ social exclusion experience, we used the 16-item Social Exclusion Experience Scale originally developed by Gilman et al. [22] and later adapted into Korean by Lee et al. [23]. The scale consists of two subscales: Ignored (e.g., “Others ignore me during conversations”; α = .95) and Excluded (e.g., “Others want to spend their leisure time with me”; α = .91). Items on the Excluded subscale are reverse-coded.

*de Jong Gierveld Loneliness Scale.* To measure participants’ loneliness, we used the 11-item scale originally developed by de Jong-Gierveld & Kamphuls [24] and later adapted into Korean by Joo & Youn [25]. The scale consists of two subscales: emotional loneliness (e.g., “I experience an empty void.”; α = .83) and social loneliness (e.g., “I always have someone to talk to about everyday problems”; α = .90). Items on the social loneliness subscale are reverse-coded.

*Perceived credibility.* Perceived credibility was assessed by asking participants to rate each of the four opinions on a 7-point Likert scale (1 = not at all credible, 7 = very credible). Ratings were averaged within each trial to create a composite credibility score, which was used in subsequent analyses. Internal consistency was high across task conditions (αs = .90–.91).

### Procedure

The task comprised four judgment conditions created by crossing task type (objective vs. subjective) with task domain (human-related vs. non-human-related). Each condition was presented twice with similar stimuli, yielding a total of eight trials. Trial order was counterbalanced using a Latin square design (see Table 1). Objective tasks involved relatively measurable or externally verifiable judgments, whereas subjective tasks involved interpretation-based evaluations without a single correct answer. In addition, tasks were categorized as human-related or non-human-related depending on whether the stimuli primarily required judgments about human characteristics or non-human objects.

**Table 1:** Overview of Judgment Task conditions and Example Items.

Task Type	Example Item	Response Scale (Likert)
Subjective, Human-related	“How would you describe the personality of the person in the image?”	1 = Focused on a single task, 10 = Multitasking
	“How would you characterize the person’s decision style?”	1 = Cautious, 10 = Flexible
Subjective, non-human-related	“Which theme best represents this artwork?”	1 = Slow-paced, 10 = Fast-paced
	“Is the artwork more practice-oriented or theory-oriented?”	1 = Practice-oriented, 10 = Theory-oriented
Objective, Human-related	“What is the estimated average stride length of the person in the photo (cm)?”	1 = 70 cm, 10 = 160 cm
	“What is the estimated arm length of the person in the photo (cm)?”	1 = 20 cm, 10 = 80 cm
Objective, non-human-related	“How many blue shapes are shown in the image?”	1 = 100, 10 = 1000
	“How many blue triangles larger than the average red triangle are shown?”	1 = 100, 10 = 1000

**Note.** Trial order was counterbalanced using a Latin square design. Full opinion stimuli are provided in the Supplementary Materials.

Participants accessed the study via an online link using their personal devices. Upon entry, they were randomly assigned to one of eight experimental versions corresponding to different Latin square orders, which counterbalanced the presentation of task conditions.

Participants were also randomly assigned to either the AI or human opinion-source condition.

After providing informed consent, participants completed basic demographic questions, followed by measures of social exclusion and loneliness. They then received instructions for the judgment tasks.

Each trial consisted of three stages. First, participants viewed a stimulus and provided an initial estimate or evaluation (pre-judgment). Next, they were presented with four opinions and rated the perceived credibility of each opinion. Although the numerical content of the opinions was identical across conditions, the opinion source was manipulated: in the human condition, opinions were attributed to human names accompanied by person icons, whereas in the AI condition, opinions were attributed to AI brand names accompanied by AI service logos. Finally, participants provided a revised judgment (post-judgment). Human names were selected using common Korean first- and last-name combinations, and AI sources were represented using widely recognized AI service names to enhance familiarity and ecological plausibility across conditions.

## Results

*Preliminary analyses.* Preliminary analyses indicated no significant differences between the AI and human opinion source groups in age, social exclusion, or loneliness (all ps > .22). Further, a chi-square test revealed no significant difference in gender distribution between groups (p = .69; see Table S3 in S1 File in the Supplementary Materials).

Baseline comparisons across Latin square orders revealed a significant difference in social exclusion, *F*(3, 350) = 5.05, *p* = .002. No other baseline variables varied by order (see Table S4 in S1 File in the Supplementary Materials). Accordingly, Latin square order was included as a covariate in all mixed-effects models.

*Main effects of task characteristics and opinion source on conformity.* Linear mixed-effects models with participant-level random intercepts were used to account for repeated observations within individuals. The analyses revealed significant main effect of task type, opinion source, and perceived credibility on conformity (see Table 2). Participants showed lower conformity in subjective tasks than in objective tasks (β = −.37, p < .001). In addition, conformity was lower when opinions were provided by human sources relative to AI sources (β = −.18, p = .010). Perceived credibility positively predicted conformity (β = .25, p < .001). Neither task domain (human-related vs. non-human-related) nor social exclusion showed a significant main effect. At the fixed-effects level, the model yielded a marginal R^2^ of.121, which increased to a conditional R^2^ of.335 when random effects were included (see Table 2).

**Table 2:** Results of the linear mixed-effects model predicting conformity.

Variable	B (SE)	95% CI	β	*t*	*p*
task type (subject)	−.11 (0.01)	[-.14, -.09]	−.37	−8.48	<.001
task domain (human related)	−.01 (0.01)	[-.04, .02]	−.03	−0.73	.466
opinion source (Human)	−.05 (0.02)	[-.10, -.01]	−.18	−2.59	.010
perceived credibility	.07 (0.01)	[.05, .08]	.25	9.19	<.001
social exclusion	−.02 (0.01)	[-.05,.00]	−.06	−1.67	.096
**Random effects (SD)**					
Participant intercept	.14				
Residual	.25				
**Effects Size**					
Marginal$R^{\text{2}}$	.121				
Conditional$R^{\text{2}}$	.335				

**Note.** Latin square order was included as a covariate in all models. Full model results are reported in the Supplemental Materials (see Table S6 in S1 File in the Supplementary Materials)

*Task Type and Opinion Source Effects on Conformity.* A linear mixed-effects model examined whether task type moderated the effect of opinion source on conformity. Replicating the main effects, participants showed significantly lower conformity in subjective tasks than in objective tasks (β = −.44, p < .001), and lower conformity when opinions were provided by human sources rather than AI sources (β = −.21, p = .014). However, the interaction between opinion source and task type was not significant (β = .11, p = .238), indicating that the difference in conformity between AI and human sources did not vary across subjective and objective task contexts. At the fixed-effects level, the model yielded a marginal R^2^ of.054, which increased to a conditional R^2^ of.308 when random effects were included(see Table 3).

**Table 3:** Linear Mixed-Effects Model Predicting Conformity: Task Type and Opinion Source.

Variable	B (SE)	95% CI	β	*t*	*p*
task type (subject)	−.14 (.02)	[-.18, -.10]	−.44	−6.81	<.001
opinion source (Human)	−.06 (.03)	[-.12, -.01]	−.21	−2.47	.014
opinion source x task type	.03 (.03)	[-.02, .09]	.11	1.18	0.238
**Random effects (SD)**					
Participant intercept	.16				
Residual	.26				
**Effects Size**					
Marginal$R^{\text{2}}$	.054				
Conditional$R^{\text{2}}$	.308				

**Note.** Latin square order was included as a covariate in all models. Full model results are reported in the Supplemental Materials (see Table S8 in S1 File in the Supplementary Materials)

Although the interaction was not significant, exploratory simple comparisons revealed higher conformity to AI than to human opinions in the objective, non-human domain condition (t = 2.58, p = .010), whereas no such differences emerged in other task contexts (see Table S5 in S1 File in the Supplementary Materials).

*Social Exclusion and Opinion Source Effects on Conformity.* A linear mixed-effects model examined whether social exclusion moderated the effect of opinion source on conformity (see Table 4). Results revealed significant main effect of social exclusion (β = −.16, *p* = .003) and opinion source (β = −.14, *p* = .006). Importantly, the interaction between social exclusion and opinion source was also significant (β = .16, *p* = .025), indicating that the association between social exclusion and conformity differed by opinion source. To probe this interaction, simple slope analyses estimated the association between social exclusion and conformity separately within the AI and human opinion-source conditions. These analyses revealed that higher levels of social exclusion were associated with reduced conformity to AI opinions (b = −.07, *p* = .003), whereas conformity to human opinions was not significantly related to social exclusion (b = −.001, *p* = .949). Task type remained a significant predictor of conformity (β = −.38, *p* < .001), whereas task domain did not show a significant effect. At the fixed-effects level, the model yielded a marginal $R^{\text{2}}$ of.064, which increased to a conditional $R^{\text{2}}$ of.309 when random effects were included (see Table 4).

**Table 4:** Linear mixed-effects model predicting conformity: Social exclusion × opinion source interaction.

Variable	B (SE)	95% CI	β	*t*	*p*
social exclusion	−.07 (.02)	[-.11, -.02]	−.16	−3.00	.003
opinion source (Human)	−.19 (.07)	[-.33, -.06]	−.14	−2.77	.006
social exclusion × source	.07 (.03)	[.01, .13]	.16	2.25	.025
task type (subject)	−.12 (.01)	[-.15, -.09]	−.38	−8.66	<.001
task domain (human related)	−.01 (.01)	[-.04,.02]	−.04	−.86	.388
**Random effects (SD)**					
Participant intercept	.15				
Residual	.26				
**Effects Size**					
Marginal$R^{\text{2}}$	.064				
Conditional$R^{\text{2}}$	.309				

**Note.** Latin square order was included as a covariate in all models. Full model results are reported in the Supplemental Materials (see Table S9 in S1 File in the Supplementary Materials)

**Fig 1:**
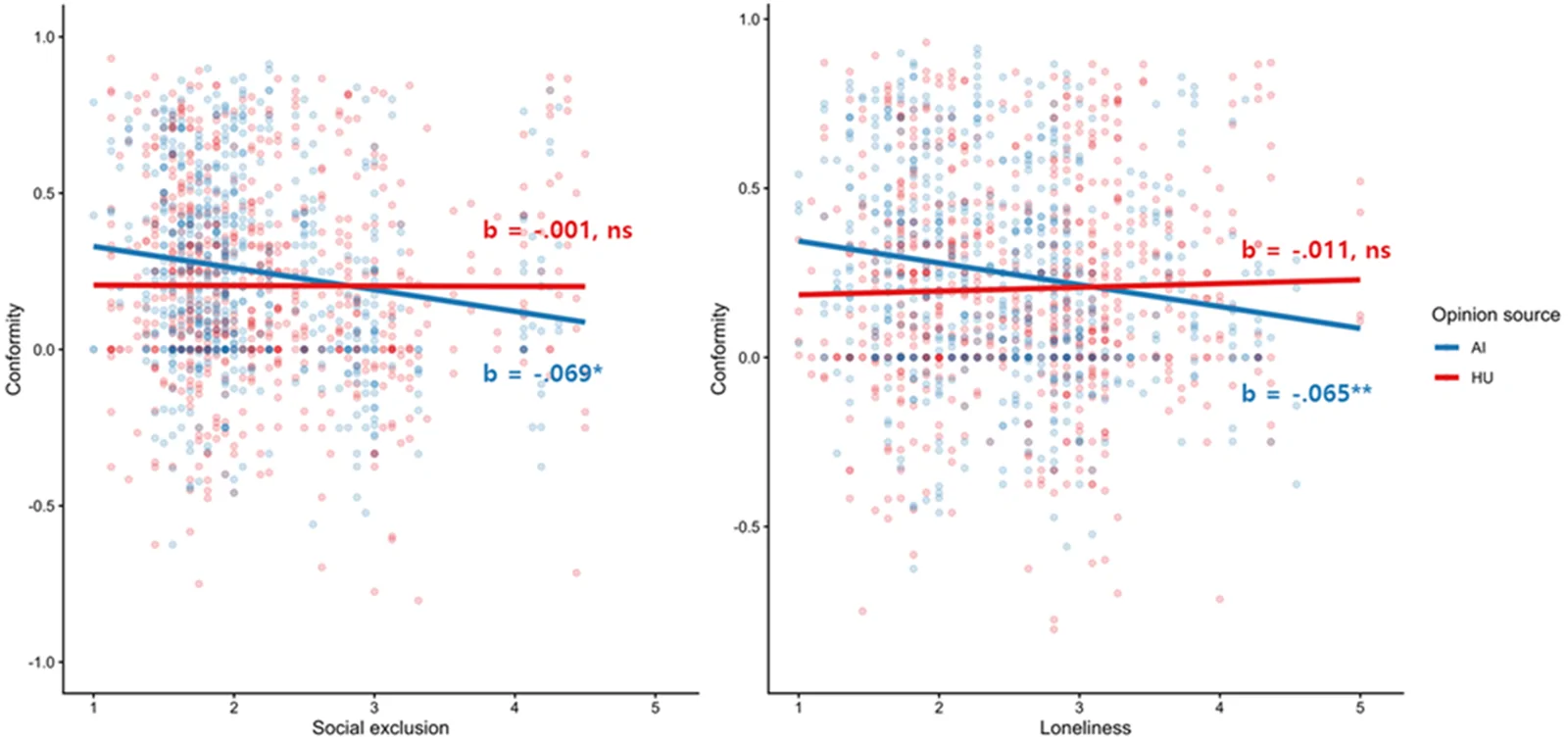
Interaction effects of social exclusion and opinion source on conformity.

A similar pattern emerged for loneliness (see Table S10 in S1 File in the Supplementary Materials). The interaction between loneliness and opinion source was significant (β = .19, *p* = .007), indicating that the association between loneliness and conformity depending on opinion source. The same procedure was applied to the loneliness × opinion source interaction. Simple slope analyses revealed that higher levels of loneliness were associated with reduced conformity to AI opinions (b = −0.06, *p* = .002), whereas conformity to human opinions was not significantly related to loneliness (b = 0.01, *p* = .553). Task type remained a significant predictor of conformity (β = −.38, *p* < .001), whereas task domain did not show a significant effect. At the fixed-effects level, the model yielded a marginal $R^{\text{2}}$ of.066, which increased to a conditional $R^{\text{2}}$ of.309 when random effects were included (see Fig 1).

## Discussion

The present research investigated how conformity is shaped by task type, opinion source (AI vs. human), and social exclusion, and whether the effects of task type and social exclusion differ across opinion sources.

Overall, participants showed lower conformity in subjective than in objective tasks and were less likely to conform to human opinions than to AI-generated opinions. This source-related effect is consistent with prior evidence of algorithm appreciation, suggesting that individuals rely more on algorithmic advice than on human input [12].

Perceived credibility of the opinion source was also associated with greater conformity, consistent with prior research emphasizing the role of source credibility in opinion susceptibility [6]. However, in the present study, social exclusion and loneliness did not show a uniform association with conformity.

The association between social exclusion and conformity differed by opinion source. Although social exclusion was not associated with overall conformity, higher levels of social disconnection predicted reduced conformity specifically to AI opinions, whereas conformity to human opinions remained largely unchanged. This pattern suggests that conformity to artificial agents does not simply increase under conditions of social disconnection.

The present findings do not necessarily contradict existing theories suggesting that individuals can respond socially to artificial agents [8,26]. Rather, they may suggest that although individuals can respond to AI in socially meaningful ways, social influence involving AI may rely less strongly on affiliative or relational motivations than traditional interpersonal social influence. Instead, responses to AI opinions may depend relatively more on informational or epistemic evaluations of the source.

Consistent with the Multi-Motive Model, responses to social exclusion depend on individuals’ appraisals of whether a given source represents a meaningful opportunity for social reconnection [19]. In this context, AI may not function as a viable social target. Instead, interactions with artificial agents may elicit greater skepticism or disengagement, resulting in reduced conformity.

The present findings tentatively suggest that conformity to AI reflects relatively greater epistemic than affiliative considerations. In contrast to conformity toward human agents, which reflects both accuracy and social alignment needs [2], participants in the present context may have treated AI more as a source of information than as a relational partner. Importantly, this distinction was particularly evident among individuals reporting higher levels of social exclusion. Although affiliative needs were not directly measured, prior research suggests that social disconnection heightens sensitivity to opportunities for meaningful reconnection [27]. In this context, artificial agents may be perceived as less capable of addressing affiliative concerns, which may contribute to the observed reduction in conformity toward AI sources. Consequently, socially excluded individuals may place less weight on AI-generated input when it fails to address affiliative concerns, contributing to the observed reduction in conformity toward artificial sources.

Notably, conformity to human opinions also did not increase under social exclusion. This result differs from prior findings showing heightened alignment with others following exclusion [16,17]. One possible explanation lies in the characteristics of the experimental context. Because the study was conducted online and the human source was represented only by a name and a pictogram, participants had limited social cues on which to base judgments about approachability or trustworthiness. Given that limited social and interpersonal information in online contexts constrains the development of benevolence-based trust [28], this lack of interpersonal information may have reduced the extent to which human sources afforded meaningful social reconnection, thereby attenuating conformity driven by affiliative motives.

Although prior work has raised concerns that individuals with low social connection may rely on AI in ways that substitute for human relationships and potentially exacerbate loneliness [29], the present findings open additional possibilities. Rather than compensating for social deficits, AI advice became less influential as social exclusion increased. This finding may be interpreted as suggesting that socially disconnected individuals do not necessarily adopt artificial agents as relational substitutes. However, because relational substitution and social approach motivations were not directly measured in the present study, this interpretation should be considered tentative. Accordingly, future research should examine how AI might function not as a replacement for human relationships, but as a potential facilitator or intermediary for social reconnection under conditions of vulnerability.

Additionally, cultural context may represent a potential boundary condition. Recent anthropomorphism research suggests that loneliness does not uniformly increase engagement with artificial agents across cultures, with some East Asian findings indicating reduced anthropomorphism under social disconnection [30,31], possibly reflecting heightened social threat sensitivity and self-protective avoidance. Although speculative, this possibility highlights the need for future research to examine whether the motivational pathways linking social exclusion to AI conformity vary across cultural contexts.

Finally, we examined whether task-related differences in conformity vary as a function of opinion source. Previous studies have shown that individuals tend to conform more to AI opinions in objective tasks, whereas they conform more to human opinions in subjective tasks [3,15]. Building on these findings, we further differentiated subjective and objective tasks according to whether they involved human-related or non-human-related content. This allowed us to examine whether task- dependent conformity to opinion sources reflects a generalizable pattern or instead depends on the social relevance of the task domain.

The interaction between task type and opinion source was not statistically significant. Nonetheless, exploratory analyses suggested that source differences emerged selectively within the objective, non-human-related task condition (e.g., dot counting), where participants showed greater conformity to AI than to human opinions, whereas no such difference appeared in objective tasks involving human-related content (e.g., arm length estimation). These patterns suggest that preferences for AI in objective domains may not generalize broadly but instead depend on the social relevance of the task context. Taken together, although participants showed overall greater conformity to AI, this effect appeared most pronounced in highly countable, non-human-related tasks. Future research should therefore examine a wider range of task conditions to more precisely delineate when AI– human differences in conformity emerge.

However, several limitations should be acknowledged when interpreting the findings. Both AI and human influence were operationalized using static opinion displays with minimal social cues, which may limit ecological validity and constrain perceptions of relational availability compared to real-world interactions. Although common Korean names and widely recognized AI service names were used to enhance familiarity and ecological plausibility across conditions, the source manipulation may still have differed on dimensions such as familiarity, visual salience, or perceived credibility beyond source type itself. In addition, although the study differentiated tasks along objective–subjective and human-related–non- human-related dimensions, the conceptual boundaries between these categories remain somewhat ambiguous, and the present task set may have varied on additional characteristics such as ambiguity, difficulty, familiarity, and social relevance. Finally, although conformity was operationalized as changes in judgments following external opinions, the present design does not allow clear differentiation between classical forms of social conformity and related processes such as advice taking, source-based trust, or heuristic reliance on perceived competence. Future research should therefore examine these processes using more naturalistic interactions, broader task contexts, and more direct assessments of the psychological mechanisms underlying conformity toward AI-generated opinions.

## Supporting information

S1 File Supplemental statistics and additional analyses. (DOCX)

S1 Dataset Anonymized participant-level dataset. (XLSX)
