# Supplementary material for: Conforming to AI vs. humans: The moderating effects of social exclusion and task type
Source: PLoS One. 2026 Aug 31;21(8):e0355478. doi: 10.1371/journal.pone.0355478 (PMC13528959; doi:10.1371/journal.pone.0355478)
Supplement: Supplementary file 1 [file pone.0355478.s001.docx]

Supplemental statics

A. Detailed statistics

**Table S1. demographic statistics**

| **Variable** | **mean (SD)** | **range** | **total** |
| --- | --- | --- | --- |
| Age | 30.54 (8.34) | 19 - 68 | N= 354 |
| gender (male) | 115 (32.49%) |  |  |
| Group (AI) | 167 (47.18%) |  |  |

**Table S2 descriptive statistics of variable**

| **Variable** | **mean (SD)** | **range** | **Cronbach alpha** |
| --- | --- | --- | --- |
| social exclusion | 2.18 ( .72) | [1.0, 4.5] | .93 |
| Loneliness | 2.51 (.78) | [1.0, 5.0] | .88 |
| Object Human related conformity | .29 (.30) | [-.58, .87] | - |
| Object non-Human related conformity | .26 (.31) | [-.62, .84] | - |
| Subject Human related conformity | .13 (.32) | [-.80, .89] | - |
| Subject non-Human related conformity | .18 (.28) | [-.70, .93] | - |
| Object Human related credibility | 3.97 (1.19) | [1.0, 6.38] | .91 |
| Object non-Human related credibility | 4.11 (1.16) | [1.0, 7.0] | .90 |
| Subject Human related credibility | 4.01 (1.21) | [1.0, 7.0] | .91 |
| Subject non-Human related credibility | 3.95 (1.20) | [1.0, 6.75] | .91 |

**Table S3 equivalence of source group**

| **Variable** | **Group** | **mean (SD)** | **t** | **mean difference** | **mean difference CI** | **p** | **hedge's g** | **hedge's g CI** |
| --- | --- | --- | --- | --- | --- | --- | --- | --- |
| Social exclusion | AI | 2.13 (.68) | -1.06 | -.08 | [-.23, .07] | .29 | -.11 | [-.32, .10] |
|  | Human | 2.21 (.75) |  |  |  |  |  |  |
| Loneliness | AI | 2.46 (.75) | -1.24 | -.1 | [-.26, .06] | .22 | -.13 | [-.34, .08] |
|  | Human | 2.56 (.79) |  |  |  |  |  |  |
| age | AI | 30.39 (8.43) | -.33 | -.29 | [-2.04, 1.46] | .75 | -.04 | [-.24, .17] |
|  | Human | 30.68 (8.28) |  |  |  |  |  |  |
| gender (male) | AI | 52 (31.14%) | $x^{2}(1)$ = .16, p = .69 | | | | | |
|  | Human | 63 (33.69%) |  |  |  |  |  |  |

**Table S4 Comparison of Baseline Characteristics across Latin Square Orders**

| **Variable** | **Group (latin sqare)** | **mean (SD)** | **F** | **P** | $\eta^{2}$ | **PostHoc (scheffe)** |
| --- | --- | --- | --- | --- | --- | --- |
| social exclusion | G1 | 2.40 (.82) | F(3, 350) = 5.05 | .002 | .041 | G1 > G4 |
|  | G2 | 2.15 (.69) |  |  |  |  |
|  | G3 | 2.13 (.73) |  |  |  |  |
|  | G4 | 2.02 (.58) |  |  |  |  |
| Loneliness | G1 | 2.67 (.75) | F(3, 350) = 2.12 | .097 | .018 | - |
|  | G2 | 2.51 (.84) |  |  |  |  |
|  | G3 | 2.44 (.78) |  |  |  |  |
|  | G4 | 2.41 (.73) |  |  |  |  |
| age | G1 | 29.85 (7.38) | F(3, 350) = 2.06 | .106 | .017 | - |
|  | G2 | 30.20 (7.40) |  |  |  |  |
|  | G3 | 32.59 (10.55) |  |  |  |  |
|  | G4 | 29.89 (7.84) |  |  |  |  |

**Table S5 t-tests for Conformity Measures by Source Type, Task type, Domain**

| **Task** | **Domain** | **opinion source** | **mean (SD)** | **t** | **Mean difference** | **Mean difference CI** | **p** | **hedge's g** | **hedge's g CI** |
| --- | --- | --- | --- | --- | --- | --- | --- | --- | --- |
| Objective | **non-human** | **AI** | **.31 (.31)** | **2.58** | **[.02, .15]** | **2.58** | **.01** | **.275** | **[.06, .49]** |
|  |  | **Human** | **.22 (.30)** |  |  |  |  |  |  |
|  | Human | AI | .30 (.30) | .68 | [-.04, .09] | .68 | .50 | .072 | [-.14, .28] |
|  |  | Human | .28 (.31) |  |  |  |  |  |  |
| Subjective | non-human | AI | .20 (.27) | .83 | [-.03, .08] | .83 | .41 | .087 | [-.12, .30] |
|  |  | Human | .17 (.29) |  |  |  |  |  |  |
|  | Human | AI | .14 (.31) | .49 | [-.05, .08] | .49 | .67 | .052 | [-.16, .26] |
|  |  | Human | .12 (.33) |  |  |  |  |  |  |

**Table S6 Results of the linear mixed-effects model predicting conformity (with social exclusion)**

| **Variable** | **B (SE)** | **β** | **t** | **p** | **95% CI** |
| --- | --- | --- | --- | --- | --- |
| **task type (subject)** | **-.11 (.01)** | **-.37** | **-8.48** | **< .001** | **[-.14, -.09]** |
| task domain (human related) | -.01 (.01) | -.03 | -.73 | .466 | [-.04, .02] |
| **opinion source (Human)** | **-.05 (.02)** | **-.18** | **-2.59** | **.01** | **[-.10, -.01]** |
| **perceived credibility** | **.07 (.01)** | **.25** | **9.19** | **< .001** | **[.05, .08]** |
| social exclusion | -.02 (.01) | -.06 | -1.67 | .096 | [-.05, .00] |
| Latin G2 | .02 (.03) | .08 | .85 | .398 | [-.03, .08] |
| Latin G3 | .04 (.03) | .13 | 1.41 | .159 | [-.02, .10] |
| **Latin G4** | **.07 (.03)** | **.24** | **2.63** | **.009** | **[.02, .13]** |
| **Random Effects (SD)** | | | | | |
| Individual | .14 | | | | |
| Residual | .25 | | | | |
| **Effects Size** | | | | | |
| $Marginal R^{2}$ | .121 | | | | |
| $Conditional R^{2}$ | .335 | | | | |

**Table S7 Results of the linear mixed-effects model predicting conformity (with Loneliness)**

| **Variable** | **B (SE)** | **β** | **t** | **p** | **95% CI** |
| --- | --- | --- | --- | --- | --- |
| **task type (subject)** | **-.11 (.01)** | **-.37** | **-8.48** | **< .001** | **[-.14, -.09]** |
| task domain (human related) | -.01 (.01) | -.03 | -.73 | .466 | [-.04, .02] |
| **opinion source (Human)** | **-.05 (.02)** | **-.18** | **-2.63** | **.009** | **[-.10, -.01]** |
| **perceived credibility** | **.07 (.01)** | **.25** | **9.19** | **< .001** | **[.05, .08]** |
| loneliness | -.02 (.01) | -.04 | -1.21 | .227 | [-.04, .01] |
| Latin G2 | .03 (.03) | .09 | .97 | .331 | [-.03, .09] |
| Latin G3 | .04 (.03) | .14 | 1.51 | .131 | [-.01, .10] |
| **Latin G4** | **.08 (.03)** | **.26** | **2.85** | **.005** | **[.03, .13]** |
| **Random Effects (SD)** | | | | | |
| Individual | .14 | | | | |
| Residual | .25 | | | | |
| **Effects Size** | | | | | |
| $Marginal R^{2}$ | .119 | | | | |
| $Conditional R^{2}$ | .335 | | | | |

**Table S8 Linear mixed-effects model predicting conformity: Task × opinion source interaction**

| **Variable** | **B (SE)** | **β** | **t** | **p** | **95% CI** |
| --- | --- | --- | --- | --- | --- |
| **task type (subject)** | **-.14 (.02)** | **-.44** | **-6.81** | **< .001** | **[-.18, -.10]** |
| opinion source (Human) | -.06 (.03) | -.21 | -2.47 | .014 | [-.12, -.01] |
| **task × source** | **.03 (.03)** | **.11** | **1.18** | **.238** | **[-.02, .09]** |
| Latin G2 | .04 (.03) | .13 | 1.26 | .208 | [-.02, .10] |
| Latin G3 | .06 (.03) | .18 | 1.82 | .069 | [-.00, .12] |
| **Latin G4** | **.10 (.03)** | **.31** | **3.27** | **.001** | **[.04, .15]** |
| **Random Effects (SD)** | | | | | |
| Individual | .16 | | | | |
| Residual | .26 | | | | |
| **Effects Size** | | | | | |
| $Marginal R^{2}$ | .054 | | | | |
| $Conditional R^{2}$ | .308 | | | | |

**Table S9 Linear mixed-effects model predicting conformity: Social exclusion × opinion source interaction**

| **Variable** | **B (SE)** | **β** | **t** | **p** | **95% CI** |
| --- | --- | --- | --- | --- | --- |
| **social exclusion** | **-.07 (.02)** | **-.16** | **-3.00** | **.003** | **[-.11, -.02]** |
| **opinion source (Human)** | **-.19 (.07)** | **-.14** | **-2.77** | **.006** | **[-.33, -.06]** |
| **social exclusion × source** | **.07 (.03)** | **.16** | **2.25** | **.025** | **[.01, .13]** |
| **task type (subject)** | **-.12 (.01)** | **-.38** | **-8.66** | **< .001** | **[-.15, -.09]** |
| task domain (human related) | -.01 (.01) | -.04 | -.86 | .388 | [-.04, .02] |
| Latin G2 | .03 (.03) | .10 | 1.05 | .297 | [-.03, .09] |
| **Latin G3** | **.05 (.03)** | **.17** | **1.7** | **.09** | **[-.01, .11]** |
| **Latin G4** | **.09 (.03)** | **.28** | **2.96** | **.003** | **[.03, .15]** |
| **Random Effects (SD)** | | | | | |
| Individual | .15 |  |  |  |  |
| Residual | .26 |  |  |  |  |
| **Effects Size** | | | | | |
| Marginal $R^{2}$ | .064 |  |  |  |  |
| Conditional $R^{2}$ | .309 |  |  |  |  |

**Table S10 Linear mixed-effects model predicting conformity: Loneliness × opinion source interaction**

| **Variable** | **B (SE)** | **β** | **t** | **p** | **95% CI** |
| --- | --- | --- | --- | --- | --- |
| **loneliness** | **-.06 (.02)** | **-.16** | **-3.12** | **.002** | **[-.11, -.02]** |
| opinion source (Human) | -.23 (.07) | -.15 | -3.21 | .001 | [-.38, -.09] |
| **loneliness × source** | **.08 (.03)** | **.19** | **2.72** | **.007** | **[.02, .13]** |
| **task type (subject)** | **-.12 (.01)** | **-.38** | **-8.66** | **< .001** | **[-.15, -.09]** |
| task domain (human related) | -.01 (.01) | -.04 | -.86 | .388 | [-.04, .02] |
| Latin G2 | .04 (.03) | .12 | 1.26 | .208 | [-.02, .10] |
| Latin G3 | .05 (.03) | .16 | 1.60 | .11 | [-.01, .11] |
| **Latin G4** | **.09 (.03)** | **.3** | **3.12** | **.002** | **[.03, .15]** |
| **Random Effects (SD)** | | | | | |
| Individual | .15 |  |  |  |  |
| Residual | .26 |  |  |  |  |
| **Effects Size** | | | | | |
| Marginal $R^{2}$ | .066 |  |  |  |  |
| Conditional $R^{2}$ | .309 |  |  |  |  |

B. additional analysis

**Table S11 repeated-measures ANOVA on perceived credibility (AI Brand)**

| **DV** | **Source brand** | **mean (SD)** | **F** | **P** | **generalized** $\eta^{2}$ | **PostHoc (scheffe)** |
| --- | --- | --- | --- | --- | --- | --- |
| credibility | Claude | 4.09 (1.01) | 9.56 | <.001 | .006 | Claude > Exaone (p = .042)  Claude > Gemini (p = .020)  Claude > GPT (p < .001) |
|  | LG Exaone | 3.97 (1.05) |  |  |  |  |
|  | Gemini | 3.95 (1.09) |  |  |  |  |
|  | chat GPT | 3.86 (1.05) |  |  |  |  |

Repeated measures ANOVA was conducted to examine differences in perceived credibility across AI brands. Results indicated a significant main effect of source brand on credibility, F = 9.56, p < .001, generalized η² = .006. Post hoc Scheffé tests revealed that Claude (M = 4.09, SD = 1.01) was rated as significantly more credible than LG Exaone (M = 3.97, SD = 1.05, p = .042), Gemini (M = 3.95, SD = 1.09, p = .020), and ChatGPT (M = 3.86, SD = 1.05, p < .001). No other pairwise differences were significant.
