# Supplementary material for: Conforming to AI vs. humans: The moderating effects of social exclusion and task type
Source: PLoS One. 2026 Aug 31;21(8):e0355478. doi: 10.1371/journal.pone.0355478 (PMC13528959; doi:10.1371/journal.pone.0355478)
Supplement: Supplementary file 2 [file pone.0355478.s004.docx]

[Journal Requirement]

**Journal Requirement #1:**
“Please ensure that your manuscript meets PLOS ONE's style requirements, including those for file naming.”

**Response:**
Thank you for this note. We have revised the manuscript and submission files to conform to the PLOS ONE style and formatting requirements, including file naming conventions.

**Journal Requirement #2:** Removal and anonymization of potentially identifying participant information in the shared dataset.

**Response:**
Thank you for this important comment. We carefully reviewed both the uploaded Supporting Information dataset and the OSF-hosted data files to ensure compliance with PLOS ONE’s data-sharing and participant privacy guidelines. Specifically, participant ID variables were removed, and exact age information was recorded into age-group categories to reduce the risk of participant identification. These anonymization procedures were applied consistently across both the supplementary dataset and the OSF repository. We also confirmed that no identifying information remained in hidden spreadsheet columns. A fully anonymized version of the dataset has now been re-uploaded.

**Journal Requirement #3**: Provision of an English-language version of the shared dataset.

**Response:**
Thank you for this comment. We revised the shared dataset to ensure that all variables and response values are presented in English. Specifically, Korean response texts that appeared in the informed consent and personal information agreement variables were converted into numeric codes following an English coding scheme (e.g., 1 = agree, 2 = disagree). We also reviewed the dataset to ensure consistency with PLOS ONE’s Data Availability guidelines and uploaded the revised English-language version accordingly.

**Journal Requirement #4:** Placement of the ethics statement only within the Methods section.

**Response:**
Thank you for this comment. We reviewed the manuscript and ensured that the ethics statement appears only in the Methods section, in accordance with PLOS ONE guidelines. Any duplicate ethics-related statements appearing elsewhere in the manuscript were removed.

**Journal Requirement #5**: Inclusion of captions for Supporting Information files at the end of the manuscript and revision of corresponding in-text citations.

**Response**:
Thank you for this comment. We added captions for all Supporting Information files at the end of the manuscript following the References section, in accordance with PLOS ONE guidelines. We also revised the corresponding in-text citations and updated the supporting file names to ensure consistency between the manuscript text and the submitted Supporting Information files.

**Journal Requirement #6**: Review and evaluation of reviewer-recommended references.

**Response:**
Thank you for this comment. We carefully reviewed the references and related literature suggested in the reviewer comments and incorporated relevant citations where appropriate. We also evaluated the suggested works in relation to the theoretical framing and interpretation of the present findings.

[Revier 1]

**Reviewer #1, Comment 1**: The theoretical frameworks were not sufficiently integrated into explicit hypotheses, and the manuscript did not clearly explain the distinct roles of each framework in predicting AI versus human conformity. The reviewer also requested clearer hypotheses regarding the main effect of opinion source, the task type × source interaction, and the social exclusion × source interaction.

**Response:** Thank you for this thoughtful and constructive comment. We agree that the original manuscript did not sufficiently clarify how the different theoretical frameworks informed the predicted patterns of conformity toward AI versus human opinions. In response, we revised the final section of the Introduction to more explicitly articulate the theoretical expectations underlying the present study. Specifically, we clarified how prior research on AI reliance in objective task contexts informed expectations regarding task type differences, and how the Multi-Motive Model informed expectations regarding the differential association between social exclusion and conformity depending on opinion source. We also revised the Introduction to more clearly explain the conceptual roles of the different theoretical frameworks in motivating the examination of opinion source effects, task type × source interactions, and social exclusion × source interactions (see Introduction, pp. 6).

**Reviewer #1, Comment 2:** The title’s use of “social needs” was considered overly broad, given that the study specifically measured self-reported social exclusion and loneliness rather than social needs as a general motivational construct or experimentally manipulated variable.

**Response:** We appreciate the reviewer’s thoughtful comment regarding the conceptual breadth of the term “social needs.” We agree that the original title could imply a broader motivational construct than was directly assessed in the present study. Because the study specifically measured participants’ self-reported social exclusion experiences, with loneliness examined in supplementary analyses, rather than social needs as a general construct or experimentally manipulated variable, we revised the title to more accurately reflect the primary moderator examined in the study. Accordingly, the title has been changed from *“Conforming to AI vs. Humans: The Moderating Effects of Social Needs and Task Type”* to *“Conforming to AI vs. Humans: The Moderating Effects of Social Exclusion and Task Type.* (see Title)”

**Reviewer #1, Comment 3:** The procedure and conformity calculation were not sufficiently clear regarding how the four presented opinions were incorporated into the single reference value (R) used in the conformity formula. The reviewer requested clarification on whether the opinions were identical, averaged, sequentially presented, or otherwise combined.

**Response:**

Thank you for this important clarification request. We revised the Method section to more clearly explain how the reference value (M) was calculated in the conformity index. Specifically, we clarified that M represents the mean value of the four presented opinions within each trial (see Method, pp. 7).

**Reviewer #1, Comment 4:** The reviewer questioned whether the task categories (objective vs. subjective; human-related vs. non-human-related) were sufficiently justified and psychologically comparable across conditions. The reviewer also requested clearer evidence or rationale that the tasks reflected the intended conceptual distinctions.

**Response:** We appreciate the reviewer’s thoughtful comment regarding the conceptual clarity and comparability of the task categories. The present task classifications were based on prior literature distinguishing objective versus subjective judgment contexts in AI–human evaluation research (e.g., Castelo et al., 2019; Hertz & Wiese, 2018; Riva et al., 2022). However, we agree that the current task set may have differed along multiple dimensions beyond the intended classifications, including ambiguity, perceptual difficulty, familiarity, social relevance, and scale interpretability. We also acknowledge that tasks grouped within the same category may not necessarily have involved identical cognitive or perceptual processes.

In response to the reviewer’s comment, we have revised the Discussion section to clarify that the present task structure should be interpreted as an operational approximation rather than a fully validated manipulation of task type. We additionally expanded the limitations section to explicitly acknowledge the potential overlap of multiple task characteristics and the need for future research to more systematically validate task constructions and directly assess participants’ perceptions of task characteristics (see Discussion, pp. 17).

**Reviewer #1, Comment 5**: The reviewer noted that the manuscript’s argument that conformity to AI may reflect epistemic rather than affiliative processes was theoretically underdeveloped. Specifically, the reviewer requested a clearer discussion of how AI conformity relates to classical theories of conformity as a fundamentally social process, and whether conformity to AI should be interpreted as traditional social influence or as a partially distinct process more closely related to advice taking, source-based trust, or heuristic reliance on perceived computational competence. The reviewer suggested that the Discussion section should engage more directly with these theoretical distinctions to better articulate the manuscript’s conceptual contribution.

Response:
Thank you for this thoughtful and theoretically important comment. We agree that the present design does not allow a definitive distinction between classical forms of social conformity and related processes such as advice taking or source-based trust. At the same time, because the present study examined the moderating roles of social exclusion and loneliness, we believe the observed responses are still meaningfully related to socially motivated processes. To address this issue more carefully, we added an additional limitation acknowledging that conformity toward AI-generated opinions may partially overlap with advice taking, source-based trust, or heuristic reliance on perceived competence, and that future research should more directly examine the underlying psychological mechanisms and social motivations involved in AI conformity (see Abstract, pp. 2 and Discussion, pp. 17-18).

**Reviewer #1, Comment 6:** The reviewer requested clearer reconciliation between the present findings and the CASA framework, as well as the broader literature on algorithm appreciation and aversion. Specifically, the reviewer noted that although CASA suggests that people may respond socially to AI, the reduced conformity toward AI under high social exclusion implies that AI may not function as a fully socially equivalent target under those conditions.

**Response:**
Thank you for this insightful comment. We revised the Discussion to more explicitly address the relationship between the present findings and the CASA framework, as well as the broader literature on algorithm appreciation and aversion. Specifically, we clarified that the present findings do not necessarily contradict prior research suggesting that individuals can respond socially to artificial agents. Instead, the results may indicate that although AI can elicit socially meaningful responses, AI may not function as a fully socially equivalent relational target under conditions of social disconnection. We further discussed the possibility that social influence involving AI may rely less strongly on affiliative motivations and relatively more on informational or epistemic evaluations of the source (see Discussion, pp. 14).

[Revier 2]

**Reviewer #2, Comment 1**: The reviewer noted that the manuscript makes relatively strong theoretical claims that conformity to AI reflects epistemic rather than affiliative motivations, despite these motivational processes not being directly measured. The reviewer suggested that these interpretations should be presented more cautiously as possible explanations rather than as demonstrated mechanisms.

**Response:**
Thank you for this important comment. We agree that the original manuscript presented overly strong interpretations regarding the motivational mechanisms underlying conformity toward AI, despite affiliative and epistemic motivations not being directly measured in the present study. In response, we revised the Discussion to adopt a more cautious interpretive tone throughout. Specifically, we reframed these claims as tentative interpretations rather than demonstrated mechanisms by incorporating more qualified language (e.g., “may suggest,” “may reflect,” and “may involve”) and by explicitly acknowledging that the present findings do not directly establish the underlying motivational processes (see Abstract, pp. 2 and Discussion pp.14-15).

**Reviewer #2, Comment 2**: The reviewer requested clearer justification for the task classifications used in the study. Specifically, the reviewer noted that the conceptual distinctions between objective versus subjective tasks and human-related versus non-human-related tasks were not sufficiently clear and asked for additional explanation regarding why the selected tasks represented these intended categories.

**Response:**
Thank you for this helpful comment. We revised the Method section to provide clearer justification for the task classifications used in the study. Specifically, we added additional explanation describing how objective tasks were intended to involve relatively measurable or externally verifiable judgments, whereas subjective tasks involved interpretation-based evaluations without a single correct answer. We also clarified that tasks were categorized as human-related or non-human-related based on whether the stimuli primarily involved judgments about human characteristics or non-human objects. In addition, we further acknowledged in the limitations section that the conceptual boundaries between these task categories may not have been perfectly distinct and that future research should more directly assess participants’ perceptions of task characteristics (see Procedure, pp. 8 and Discussion, pp. 17).

**Reviewer #2, Comment 3:** The reviewer noted that the source manipulation may involve potential confounds beyond source type itself. Specifically, the reviewer suggested that the comparison between human names with person icons and AI brand names with AI logos may also differ in familiarity, perceived credibility, or visual salience, and requested either a more explicit discussion of these limitations or additional manipulation-check evidence if available.

**Response:**
Thank you for this important comment. We agree that the source manipulation may involve characteristics beyond source type itself, including familiarity, perceived credibility, and visual salience. In response, we revised the Method section to clarify that common Korean names and widely recognized AI service names were selected to enhance familiarity and ecological plausibility across conditions. In addition, we added a corresponding limitation acknowledging that the source manipulation may still have differed on dimensions beyond source type alone and that future research should more carefully disentangle these factors through more controlled manipulations and direct manipulation checks (see Procedure, pp. 9 and Discussion pp.17).

**Reviewer #2, Comment 4:** The reviewer requested more complete reporting of the statistical analyses. Specifically, the reviewer asked for clearer description of the mixed-effects model specification, including the random-effects structure, treatment of continuous predictors, details of the simple slope analyses, and clarification regarding how potential edge cases in the conformity index were handled.

**Response:**
Thank you for this helpful comment. We revised the Results section to provide clearer information regarding the mixed-effects model specification. Specifically, we clarified that the analyses were conducted using linear mixed-effects models including participant-level random intercepts to account for repeated observations within individuals (see Result, pp. 10).

**Reviewer #2, Comment 5:** The reviewer suggested that the manuscript’s conclusions should remain more closely tied to the data. Specifically, the reviewer noted that while the findings support differential associations between social exclusion and conformity depending on opinion source, broader claims regarding AI as a relational substitute or AI conformity as primarily epistemic should be presented more cautiously.

**Response:**
Thank you for this thoughtful comment. We agree that some interpretations in the original manuscript extended beyond what could be directly concluded from the present data. In response, we revised the Discussion and Conclusion sections to ensure that the interpretations remain more closely tied to the observed findings. Specifically, we toned down broader claims regarding AI as a relational substitute and the epistemic nature of AI conformity by using more cautious and qualified language throughout the manuscript (see Discussion, pp. 17-18).

**Reviewer #2, Comment 6**: The reviewer noted that although the manuscript was generally understandable, several awkward or ungrammatical expressions reduced clarity and readability. The reviewer recommended careful English editing throughout the manuscript and provided examples of specific phrases that should be revised to more natural academic English.

**Response:**

Thank you for this helpful comment. We carefully reviewed the manuscript and revised a number of awkward or ungrammatical expressions throughout the text to improve clarity and readability. We also corrected the specific examples noted by the reviewer and conducted additional language editing to improve overall academic English style and expression.
