# Supplementary material for: Conforming to AI vs. humans: The moderating effects of social exclusion and task type
Source: PLoS One. 2026 Aug 31;21(8):e0355478. doi: 10.1371/journal.pone.0355478 (PMC13528959; doi:10.1371/journal.pone.0355478)
Supplement: Supplementary file 3 [file pone.0355478.s005.docx]

Response

Reviewer 2.

**Review 1**

The reviewer requested additional statistical clarifications. Specifically, the reviewer asked us to clarify whether continuous predictors were centered or standardized, how the simple slope analyses were conducted, whether random slopes were considered, and how potential edge cases in the conformity index were handled, particularly cases in which the denominator could equal zero.

Response
Thank you for requesting these additional statistical clarifications. We have now clarified these points in the revised manuscript.

First, continuous predictors were entered into the models without mean-centering. Standardized regression coefficients (β) were reported to facilitate interpretation and comparison across predictors.

Second, we clarified how the simple slope analyses were conducted. To probe the significant interaction between social exclusion and opinion source, we estimated the simple effect of social exclusion on conformity separately within the AI and human opinion-source conditions. The same procedure was applied to the loneliness × opinion source interaction.

Third, regarding the random-effects structure, random slopes were not specified or estimated in the final models. The mixed-effects models included participant-level random intercepts to account for repeated observations nested within participants. Thus, the random-effects structure used in the analyses was a random-intercept-only structure.

Finally, we clarified the issue of potential edge cases in the conformity index. In the present study, the denominator of the conformity index could not equal zero by design. Specifically, the reference values used in the conformity calculation were decimal values, whereas participants’ responses were restricted to integer Likert-type values. Therefore, participants’ initial responses could not be identical to the reference value used in the denominator.

**Review 2**

The reviewer noted that the statement that the findings “indicate” socially disconnected individuals may not readily adopt AI as a relational substitute was stronger than warranted by the present data. Because relational substitution and social approach motivations were not directly measured, the reviewer asked us to present this point as a possible interpretation rather than as a conclusion directly established by the study.

Response
Thank you for this helpful suggestion. We agree that the previous wording was stronger than warranted because the present study did not directly measure relational substitution or social approach motivations. We have therefore softened this claim in the revised manuscript. Specifically, we revised the statement to frame it as a possible interpretation rather than as a conclusion directly established by the data. The revised text now states that one possible interpretation is that socially disconnected individuals may not necessarily adopt artificial agents as relational substitutes, while also noting that this interpretation remains tentative and should be examined more directly in future research.

**Review 3**

The reviewer requested a final language check and identified several remaining grammatical issues, including the phrases “a significant main effects,” “the relationship between social exclusion and conformity depending on opinion source,” and the corresponding sentence regarding loneliness.

Response

Thank you for identifying these remaining language issues. We have corrected “a significant main effects” to “a significant main effect.” We also revised the sentence concerning social exclusion to state that “the association between social exclusion and conformity differed by opinion source,” and corrected the corresponding sentence regarding loneliness in the same way. In addition, we conducted a final language check throughout the manuscript to improve clarity and readability.
