# Supplementary material for: Conforming to AI vs. humans: The moderating effects of social exclusion and task type
Source: PLoS One. 2026 Aug 31;21(8):e0355478. doi: 10.1371/journal.pone.0355478 (PMC13528959; doi:10.1371/journal.pone.0355478)
Supplement: Peer review history

# Peer review: Decision Letter 0

**DOI:** 10.1371/journal.pone.0355478.r001

**Authors:** Lianshan Zhang

6 May 2026

Dear Dr. Lee,

Thank you for submitting your manuscript to PLOS ONE. After careful consideration, we feel that it has merit but does not fully meet PLOS ONE’s publication criteria as it currently stands. Therefore, we invite you to submit a revised version of the manuscript that addresses the points raised during the review process.

Please submit your revised manuscript by Jun 20 2026 11:59PM**.**  If you will need more time than this to complete your revisions, please reply to this message or contact the journal office at plosone@plos.org. When you're ready to submit your revision, log on to https://www.editorialmanager.com/pone/ and select the 'Submissions Needing Revision' folder to locate your manuscript file.

- A letter that responds to each point raised by the academic editor and reviewer(s). You should upload this letter as a separate file labeled 'Response to Reviewers'.
- A marked-up copy of your manuscript that highlights changes made to the original version. You should upload this as a separate file labeled 'Revised Manuscript with Track Changes'.
- An unmarked version of your revised paper without tracked changes. You should upload this as a separate file labeled 'Manuscript'.

If you would like to make changes to your financial disclosure, please include your updated statement in your cover letter. Guidelines for resubmitting your figure files are available below the reviewer comments at the end of this letter.

If applicable, we recommend that you deposit your laboratory protocols in protocols.io to enhance the reproducibility of your results. Protocols.io assigns your protocol its own identifier (DOI) so that it can be cited independently in the future. For instructions see: https://journals.plos.org/plosone/s/submission-guidelines#loc-laboratory-protocols. Additionally, PLOS ONE offers an option for publishing peer-reviewed Lab Protocol articles, which describe protocols hosted on protocols.io. Read more information on sharing protocols at https://plos.org/protocols?utm_medium=editorial-email&utm_source=authorletters&utm_campaign=protocols.

As the corresponding author, your ORCID iD is verified in the submission system and will appear in the published article. PLOS supports the use of ORCID, and we encourage all coauthors to register for an ORCID iD and use it as well. Please encourage your coauthors to verify their ORCID iD within the submission system before final acceptance, as unverified ORCID iDs will not appear in the published article. *Only*  the individual author can complete the verification step; PLOS staff *cannot*  verify ORCID iDs on behalf of authors.

We look forward to receiving your revised manuscript.

Kind regards,

Lianshan Zhang

Academic Editor

PLOS One

Journal Requirements:

When submitting your revision, we need you to address these additional requirements.

1. Please ensure that your manuscript meets PLOS ONE's style requirements, including those for file naming. The PLOS ONE style templates can be found at

https://journals.plos.org/plosone/s/file?id=wjVg/PLOSOne_formatting_sample_main_body.pdf and

https://journals.plos.org/plosone/s/file?id=ba62/PLOSOne_formatting_sample_title_authors_affiliations.pdf

2. We note that there is identifying data in the Supporting Information file <osf uploaded file.zip>. Due to the inclusion of these potentially identifying data, we have removed this file from your file inventory. Prior to sharing human research participant data, authors should consult with an ethics committee to ensure data are shared in accordance with participant consent and all applicable local laws.

Data sharing should never compromise participant privacy. It is therefore not appropriate to publicly share personally identifiable data on human research participants. The following are examples of data that should not be shared:

-Name, initials, physical address

-Ages more specific than whole numbers

-Internet protocol (IP) address

-Specific dates (birth dates, death dates, examination dates, etc.)

-Contact information such as phone number or email address

-Location data

-ID numbers that seem specific (long numbers, include initials, titled “Hospital ID”) rather than random (small numbers in numerical order)

Data that are not directly identifying may also be inappropriate to share, as in combination they can become identifying. For example, data collected from a small group of participants, vulnerable populations, or private groups should not be shared if they involve indirect identifiers (such as sex, ethnicity, location, etc.) that may risk the identification of study participants.

Additional guidance on preparing raw data for publication can be found in our Data Policy (https://journals.plos.org/plosone/s/data-availability#loc-human-research-participant-data-and-other-sensitive-data) and in the following article: http://www.bmj.com/content/340/bmj.c181.long.

Please remove or anonymize all personal information (Age), ensure that the data shared are in accordance with participant consent, and re-upload a fully anonymized data set. Please note that spreadsheet columns with personal information must be removed and not hidden as all hidden columns will appear in the published file.

3. Thank you for providing your underlying data as Supporting Information.

We note that the data set contains text or data that is not in English. Please note that PLOS is an English-language publisher, so we require data sets to be provided in English as well. Please upload an English-language version of your data set.

This will also allow us to determine if your data follows PLOS standards per our Data Availability policy here: https://journals.plos.org/plosone/s/data-availability

4. Your ethics statement should only appear in the Methods section of your manuscript. If your ethics statement is written in any section besides the Methods, please move it to the Methods section and delete it from any other section. Please ensure that your ethics statement is included in your manuscript, as the ethics statement entered into the online submission form will not be published alongside your manuscript.

5. Please include captions for your Supporting Information files at the end of your manuscript, and update any in-text citations to match accordingly. Please see our Supporting Information guidelines for more information: http://journals.plos.org/plosone/s/supporting-information.

6. If the reviewer comments include a recommendation to cite specific previously published works, please review and evaluate these publications to determine whether they are relevant and should be cited. There is no requirement to cite these works unless the editor has indicated otherwise.

[Note: HTML markup is below. Please do not edit.]

Reviewers' comments:

Reviewer's Responses to Questions

**Comments to the Author**

1. Is the manuscript technically sound, and do the data support the conclusions?

Reviewer #1: Yes

Reviewer #2: Yes

**********

2. Has the statistical analysis been performed appropriately and rigorously?

Reviewer #1: Yes

Reviewer #2: Yes

**********

3. Have the authors made all data underlying the findings in their manuscript fully available?

The PLOS Data policy

Reviewer #1: Yes

Reviewer #2: Yes

**********

4. Is the manuscript presented in an intelligible fashion and written in standard English?

Reviewer #1: Yes

Reviewer #2: Yes

**********

Reviewer #1: Thank you for the opportunity to review this manuscript. The paper examines whether conformity to AI versus human opinions is shaped by task type and by participants’ social exclusion experiences, using an online experiment with Korean adults. The topic is timely, and the attempt to compare conformity toward artificial and human sources within one design is valuable. The manuscript is also interesting because it combines source effects, task characteristics, and individual differences rather than treating conformity to AI as a simple extension of traditional conformity research. At the same time, the current version still has several important conceptual, methodological, and interpretive problems that need substantial revision before the manuscript is ready for publication.

Introduction

1.The manuscript draws on Advice Response Theory, CASA, algorithm aversion/appreciation, and the Multi-Motive Model, but these perspectives are not translated into explicit hypotheses in a systematic way. As written, the paper moves from broad theory to empirical tests without clearly stating which framework is primary, what each framework uniquely predicts, and why task type and social exclusion should operate differently across AI and human sources. The study would be much stronger if the authors formulated explicit hypotheses for the source main effect, the task type by source interaction, and the social exclusion by source interaction, rather than leaving the reader to infer the logic from the literature review.

2. The title refers to “social needs,” which implies a broad motivational construct, but the study actually measures self-reported social exclusion experience and later discusses loneliness as a related moderator. These are not the same as social needs in a general sense, and they are not experimentally manipulated.

Method

1. The manuscript states that each trial includes four opinions that participants rate for credibility, yet the conformity formula uses a single reference value, R. It is therefore unclear whether the four opinions were numerically identical, averaged into one reference score, presented sequentially, or somehow collapsed in another way.

2. The task construction also needs stronger justification. The study crosses objective versus subjective tasks with human-related versus non-human-related domains, but the sample items suggest that these categories may differ on several dimensions at once, including ambiguity, perceptual difficulty, familiarity, social relevance, and scale interpretability. For example, estimating stride length and counting shapes are both labeled objective, but they likely involve very different levels of uncertainty and cognitive process. Likewise, judging personality from an image and interpreting artwork are both labeled subjective, but they may not be psychologically comparable. Because the theoretical argument depends heavily on task structure, the manuscript should provide evidence that these task categories were validated or at least perceived by participants in the intended way.

Discussion

1. At present, the manuscript argues that conformity to AI may be driven more by epistemic considerations than by affiliative motivations, but this theoretical move is not developed in enough depth. The authors should more clearly explain how this finding speaks to, extends, or challenges the classical view of conformity as a fundamentally social process. In particular, the manuscript should directly address whether conformity to AI is still a form of social influence in the traditional sense, or whether it reflects a partially different process that is closer to advice taking, source-based trust, or heuristic deference to perceived computational competence. Without a stronger theoretical dialogue on this point, the Discussion remains somewhat descriptive and does not fully capitalize on the manuscript’s conceptual potential.

2. The manuscript would also benefit from a more explicit reconciliation of the current findings with the CASA framework and with the broader literature on algorithm appreciation and algorithm aversion. The paper cites CASA to justify why people may respond socially to AI, yet the results seem to suggest an important boundary condition: participants may conform to AI, but this conformity appears to weaken when social exclusion is high, which implies that AI is not functioning as a socially equivalent target under those conditions. This is theoretically important, but the Discussion does not sufficiently unpack the tension.

Reviewer #2: This manuscript addresses a timely and relevant question, and the overall design is clear. The use of repeated judgment trials and mixed-effects models is appropriate for the data structure. The finding that social exclusion is associated with reduced conformity to AI, but not to human sources, is potentially interesting. However, I believe the manuscript requires substantial revision before it is ready for publication.

1. Theoretical claims should be more cautious.

The manuscript repeatedly suggests that conformity to AI reflects epistemic rather than affiliative considerations. However, these motivations were not directly measured in the present study. The current results support a moderation pattern involving self-reported social exclusion, but they do not directly establish the underlying motivational mechanism. Please tone down these claims and present them as interpretations rather than demonstrated processes.

2. The task categories need clearer justification.

The distinction between objective vs. subjective tasks and human-related vs. non-human-related tasks is interesting, but the conceptual boundaries remain somewhat unclear. Please explain more clearly why these tasks were selected and how they represent the intended categories.

3. The source manipulation may involve confounds.

The comparison between “human names + person icons” and “AI brand names + AI logos” may capture more than source type alone, including familiarity, perceived credibility, or visual salience. This issue should be discussed more explicitly, or supported with manipulation-check evidence if available.

4. The statistical reporting should be more complete.

The mixed-effects approach is appropriate, but the manuscript would benefit from clearer reporting of the model specification, including the random-effects structure, treatment of continuous predictors, and details of the simple slope analyses. It would also be helpful to clarify how the conformity index was handled in possible edge cases.

5. The conclusions should stay closer to the data.

The results support the claim that the relation between social exclusion and conformity differs by opinion source in this study. However, broader conclusions about AI not serving as a relational substitute, or about AI conformity being primarily epistemic, should be stated more cautiously.

6. The manuscript would benefit from careful English editing.

The paper is understandable overall, but there are several awkward or ungrammatical expressions. For example, “a attainable target” should be corrected to “an attainable target,” and “epistemic more than affiliative considerations” could be rewritten in more natural academic English, such as “epistemic rather than affiliative considerations.” Careful language editing would improve clarity and readability.

Overall, this is a promising manuscript, but I recommend major revision before it can be considered for publication.

**********

what does this mean?). If published, this will include your full peer review and any attached files.

If you choose “no”, your identity will remain anonymous but your review may still be made public.

**Do you want your identity to be public for this peer review?**  For information about this choice, including consent withdrawal, please see our Privacy Policy

Reviewer #1: No

Reviewer #2: No

**********

[NOTE: If reviewer comments were submitted as an attachment file, they will be attached to this email and accessible via the submission site. Please log into your account, locate the manuscript record, and check for the action link "View Attachments". If this link does not appear, there are no attachment files.]

To ensure your figures meet our technical requirements, please review our figure guidelines: https://journals.plos.org/plosone/s/figures

You may also use PLOS’s free figure tool, NAAS, to help you prepare publication quality figures: https://journals.plos.org/plosone/s/figures#loc-tools-for-figure-preparation.

NAAS will assess whether your figures meet our technical requirements by comparing each figure against our figure specifications.

# Author comment: Author response to Decision Letter 1

**DOI:** 10.1371/journal.pone.0355478.r002

20 May 2026

[Journal Requirement]

Journal Requirement #1:

“Please ensure that your manuscript meets PLOS ONE's style requirements, including those for file naming.”

Response:

Thank you for this note. We have revised the manuscript and submission files to conform to the PLOS ONE style and formatting requirements, including file naming conventions.

Journal Requirement #2: Removal and anonymization of potentially identifying participant information in the shared dataset.

Response:

Thank you for this important comment. We carefully reviewed both the uploaded Supporting Information dataset and the OSF-hosted data files to ensure compliance with PLOS ONE’s data-sharing and participant privacy guidelines. Specifically, participant ID variables were removed, and exact age information was recorded into age-group categories to reduce the risk of participant identification. These anonymization procedures were applied consistently across both the supplementary dataset and the OSF repository. We also confirmed that no identifying information remained in hidden spreadsheet columns. A fully anonymized version of the dataset has now been re-uploaded.

Journal Requirement #3: Provision of an English-language version of the shared dataset.

Response:

Thank you for this comment. We revised the shared dataset to ensure that all variables and response values are presented in English. Specifically, Korean response texts that appeared in the informed consent and personal information agreement variables were converted into numeric codes following an English coding scheme (e.g., 1 = agree, 2 = disagree). We also reviewed the dataset to ensure consistency with PLOS ONE’s Data Availability guidelines and uploaded the revised English-language version accordingly.

Journal Requirement #4: Placement of the ethics statement only within the Methods section.

Response:

Thank you for this comment. We reviewed the manuscript and ensured that the ethics statement appears only in the Methods section, in accordance with PLOS ONE guidelines. Any duplicate ethics-related statements appearing elsewhere in the manuscript were removed.

Journal Requirement #5: Inclusion of captions for Supporting Information files at the end of the manuscript and revision of corresponding in-text citations.

Response:

Thank you for this comment. We added captions for all Supporting Information files at the end of the manuscript following the References section, in accordance with PLOS ONE guidelines. We also revised the corresponding in-text citations and updated the supporting file names to ensure consistency between the manuscript text and the submitted Supporting Information files.

Journal Requirement #6: Review and evaluation of reviewer-recommended references.

Response:

Thank you for this comment. We carefully reviewed the references and related literature suggested in the reviewer comments and incorporated relevant citations where appropriate. We also evaluated the suggested works in relation to the theoretical framing and interpretation of the present findings.

[Revier 1]

Reviewer #1, Comment 1: The theoretical frameworks were not sufficiently integrated into explicit hypotheses, and the manuscript did not clearly explain the distinct roles of each framework in predicting AI versus human conformity. The reviewer also requested clearer hypotheses regarding the main effect of opinion source, the task type × source interaction, and the social exclusion × source interaction.

Response: Thank you for this thoughtful and constructive comment. We agree that the original manuscript did not sufficiently clarify how the different theoretical frameworks informed the predicted patterns of conformity toward AI versus human opinions. In response, we revised the final section of the Introduction to more explicitly articulate the theoretical expectations underlying the present study. Specifically, we clarified how prior research on AI reliance in objective task contexts informed expectations regarding task type differences, and how the Multi-Motive Model informed expectations regarding the differential association between social exclusion and conformity depending on opinion source. We also revised the Introduction to more clearly explain the conceptual roles of the different theoretical frameworks in motivating the examination of opinion source effects, task type × source interactions, and social exclusion × source interactions (see Introduction, pp. 6).

Reviewer #1, Comment 2: The title’s use of “social needs” was considered overly broad, given that the study specifically measured self-reported social exclusion and loneliness rather than social needs as a general motivational construct or experimentally manipulated variable.

Response: We appreciate the reviewer’s thoughtful comment regarding the conceptual breadth of the term “social needs.” We agree that the original title could imply a broader motivational construct than was directly assessed in the present study. Because the study specifically measured participants’ self-reported social exclusion experiences, with loneliness examined in supplementary analyses, rather than social needs as a general construct or experimentally manipulated variable, we revised the title to more accurately reflect the primary moderator examined in the study. Accordingly, the title has been changed from “Conforming to AI vs. Humans: The Moderating Effects of Social Needs and Task Type” to “Conforming to AI vs. Humans: The Moderating Effects of Social Exclusion and Task Type. (see Title)”

Reviewer #1, Comment 3: The procedure and conformity calculation were not sufficiently clear regarding how the four presented opinions were incorporated into the single reference value (R) used in the conformity formula. The reviewer requested clarification on whether the opinions were identical, averaged, sequentially presented, or otherwise combined.

Response:

Thank you for this important clarification request. We revised the Method section to more clearly explain how the reference value (M) was calculated in the conformity index. Specifically, we clarified that M represents the mean value of the four presented opinions within each trial (see Method, pp. 7).

Reviewer #1, Comment 4: The reviewer questioned whether the task categories (objective vs. subjective; human-related vs. non-human-related) were sufficiently justified and psychologically comparable across conditions. The reviewer also requested clearer evidence or rationale that the tasks reflected the intended conceptual distinctions.

Response: We appreciate the reviewer’s thoughtful comment regarding the conceptual clarity and comparability of the task categories. The present task classifications were based on prior literature distinguishing objective versus subjective judgment contexts in AI–human evaluation research (e.g., Castelo et al., 2019; Hertz & Wiese, 2018; Riva et al., 2022). However, we agree that the current task set may have differed along multiple dimensions beyond the intended classifications, including ambiguity, perceptual difficulty, familiarity, social relevance, and scale interpretability. We also acknowledge that tasks grouped within the same category may not necessarily have involved identical cognitive or perceptual processes.

In response to the reviewer’s comment, we have revised the Discussion section to clarify that the present task structure should be interpreted as an operational approximation rather than a fully validated manipulation of task type. We additionally expanded the limitations section to explicitly acknowledge the potential overlap of multiple task characteristics and the need for future research to more systematically validate task constructions and directly assess participants’ perceptions of task characteristics (see Discussion, pp. 17).

Reviewer #1, Comment 5: The reviewer noted that the manuscript’s argument that conformity to AI may reflect epistemic rather than affiliative processes was theoretically underdeveloped. Specifically, the reviewer requested a clearer discussion of how AI conformity relates to classical theories of conformity as a fundamentally social process, and whether conformity to AI should be interpreted as traditional social influence or as a partially distinct process more closely related to advice taking, source-based trust, or heuristic reliance on perceived computational competence. The reviewer suggested that the Discussion section should engage more directly with these theoretical distinctions to better articulate the manuscript’s conceptual contribution.

Response:

Thank you for this thoughtful and theoretically important comment. We agree that the present design does not allow a definitive distinction between classical forms of social conformity and related processes such as advice taking or source-based trust. At the same time, because the present study examined the moderating roles of social exclusion and loneliness, we believe the observed responses are still meaningfully related to socially motivated processes. To address this issue more carefully, we added an additional limitation acknowledging that conformity toward AI-generated opinions may partially overlap with advice taking, source-based trust, or heuristic reliance on perceived competence, and that future research should more directly examine the underlying psychological mechanisms and social motivations involved in AI conformity (see Abstract, pp. 2 and Discussion, pp. 17-18).

Reviewer #1, Comment 6: The reviewer requested clearer reconciliation between the present findings and the CASA framework, as well as the broader literature on algorithm appreciation and aversion. Specifically, the reviewer noted that although CASA suggests that people may respond socially to AI, the reduced conformity toward AI under high social exclusion implies that AI may not function as a fully socially equivalent target under those conditions.

Response:

Thank you for this insightful comment. We revised the Discussion to more explicitly address the relationship between the present findings and the CASA framework, as well as the broader literature on algorithm appreciation and aversion. Specifically, we clarified that the present findings do not necessarily contradict prior research suggesting that individuals can respond socially to artificial agents. Instead, the results may indicate that although AI can elicit socially meaningful responses, AI may not function as a fully socially equivalent relational target under conditions of social disconnection. We further discussed the possibility that social influence involving AI may rely less strongly on affiliative motivations and relatively more on informational or epistemic evaluations of the source (see Discussion, pp. 14).

[Revier 2]

Reviewer #2, Comment 1: The reviewer noted that the manuscript makes relatively strong theoretical claims that conformity to AI reflects epistemic rather than affiliative motivations, despite these motivational processes not being directly measured. The reviewer suggested that these interpretations should be presented more cautiously as possible explanations rather than as demonstrated mechanisms.

Response:

Thank you for this important comment. We agree that the original manuscript presented overly strong interpretations regarding the motivational mechanisms underlying conformity toward AI, despite affiliative and epistemic motivations not being directly measured in the present study. In response, we revised the Discussion to adopt a more cautious interpretive tone throughout. Specifically, we reframed these claims as tentative interpretations rather than demonstrated mechanisms by incorporating more qualified language (e.g., “may suggest,” “may reflect,” and “may involve”) and by explicitly acknowledging that the present findings do not directly establish the underlying motivational processes (see Abstract, pp. 2 and Discussion pp.14-15).

Reviewer #2, Comment 2: The reviewer requested clearer justification for the task classifications used in the study. Specifically, the reviewer noted that the conceptual distinctions between objective versus subjective tasks and human-related versus non-human-related tasks were not sufficiently clear and asked for additional explanation regarding why the selected tasks represented these intended categories.

Response:

Thank you for this helpful comment. We revised the Method section to provide clearer justification for the task classifications used in the study. Specifically, we added additional explanation describing how objective tasks were intended to involve relatively measurable or externally verifiable judgments, whereas subjective tasks involved interpretation-based evaluations without a single correct answer. We also clarified that tasks were categorized as human-related or non-human-related based on whether the stimuli primarily involved judgments about human characteristics or non-human objects. In addition, we further acknowledged in the limitations section that the conceptual boundaries between these task categories may not have been perfectly distinct and that future research should more directly assess participants’ perceptions of task characteristics (see Procedure, pp. 8 and Discussion, pp. 17).

Reviewer #2, Comment 3: The reviewer noted that the source manipulation may involve potential confounds beyond source type itself. Specifically, the reviewer suggested that the comparison between human names with person icons and AI brand names with AI logos may also differ in familiarity, perceived credibility, or visual salience, and requested either a more explicit discussion of these limitations or additional manipulation-check evidence if available.

Response:

Thank you for this important comment. We agree that the source manipulation may involve characteristics beyond source type itself, including familiarity, perceived credibility, and visual salience. In response, we revised the Method section to clarify that common Korean names and widely recognized AI service names were selected to enhance familiarity and ecological plausibility across conditions. In addition, we added a corresponding limitation acknowledging that the source manipulation may still have differed on dimensions beyond source type alone and that future research should more carefully disentangle these factors through more controlled manipulations and direct manipulation checks (see Procedure, pp. 9 and Discussion pp.17).

Reviewer #2, Comment 4: The reviewer requested more complete reporting of the statistical analyses. Specifically, the reviewer asked for clearer description of the mixed-effects model specification, including the random-effects structure, treatment of continuous predictors, details of the simple slope analyses, and clarification regarding how potential edge cases in the conformity index were handled.

Response:

Thank you for this helpful comment. We revised the Results section to provide clearer information regarding the mixed-effects model specification. Specifically, we clarified that the analyses were conducted using linear mixed-effects models including participant-level random intercepts to account for repeated observations within individuals (see Result, pp. 10).

Reviewer #2, Comment 5: The reviewer suggested that the manuscript’s conclusions should remain more closely tied to the data. Specifically, the reviewer noted that while the findings support differential associations between social exclusion and conformity depending on opinion source, broader claims regarding AI as a relational substitute or AI conformity as primarily epistemic should be presented more cautiously.

Response:

Thank you for this thoughtful comment. We agree that some interpretations in the original manuscript extended beyond what could be directly concluded from the present data. In response, we revised the Discussion and Conclusion sections to ensure that the interpretations remain more closely tied to the observed findings. Specifically, we toned down broader claims regarding AI as a relational substitute and the epistemic nature of AI conformity by using more cautious and qualified language throughout the manuscript (see Discussion, pp. 17-18).

Reviewer #2, Comment 6: The reviewer noted that al

Attachment Submitted filename: Response to Reviewers.docx

# Peer review: Decision Letter 1

**DOI:** 10.1371/journal.pone.0355478.r003

**Authors:** Lianshan Zhang

24 Jun 2026

Dear Dr. Lee,

Please submit your revised manuscript by **Aug 08 2026 11:59PM.**  If you will need more time than this to complete your revisions, please reply to this message or contact the journal office at plosone@plos.org. When you're ready to submit your revision, log on to https://www.editorialmanager.com/pone/ and select the 'Submissions Needing Revision' folder to locate your manuscript file.

- A letter that responds to each point raised by the academic editor and reviewer(s). You should upload this letter as a separate file labeled 'Response to Reviewers'.
- A marked-up copy of your manuscript that highlights changes made to the original version. You should upload this as a separate file labeled 'Revised Manuscript with Track Changes'.
- An unmarked version of your revised paper without tracked changes. You should upload this as a separate file labeled 'Manuscript'.

If you would like to make changes to your financial disclosure, please include your updated statement in your cover letter. Guidelines for resubmitting your figure files are available below the reviewer comments at the end of this letter.

If applicable, we recommend that you deposit your laboratory protocols in protocols.io to enhance the reproducibility of your results. Protocols.io assigns your protocol its own identifier (DOI) so that it can be cited independently in the future. For instructions see: https://journals.plos.org/plosone/s/submission-guidelines#loc-laboratory-protocols. Additionally, PLOS ONE offers an option for publishing peer-reviewed Lab Protocol articles, which describe protocols hosted on protocols.io. Read more information on sharing protocols at https://plos.org/protocols?utm_medium=editorial-email&utm_source=authorletters&utm_campaign=protocols.

As the corresponding author, your ORCID iD is verified in the submission system and will appear in the published article. PLOS supports the use of ORCID, and we encourage all coauthors to register for an ORCID iD and use it as well. Please encourage your coauthors to verify their ORCID iD within the submission system before final acceptance, as unverified ORCID iDs will not appear in the published article. *Only*  the individual author can complete the verification step; PLOS staff *cannot*  verify ORCID iDs on behalf of authors.

We look forward to receiving your revised manuscript.

Kind regards,

Lianshan Zhang

Academic Editor

PLOS One

Journal Requirements:

If the reviewer comments include a recommendation to cite specific previously published works, please review and evaluate these publications to determine whether they are relevant and should be cited. There is no requirement to cite these works unless the editor has indicated otherwise.

Please review your reference list to ensure that it is complete and correct. If you have cited papers that have been retracted, please include the rationale for doing so in the manuscript text, or remove these references and replace them with relevant current references. Any changes to the reference list should be mentioned in the rebuttal letter that accompanies your revised manuscript. If you need to cite a retracted article, indicate the article’s retracted status in the References list and also include a citation and full reference for the retraction notice.

**Additional Editor Comments:**

1. Please carefully check that all claims and conclusions are fully supported by the data and that the findings are not overstated. This is particularly important for PLOS ONE, where conclusions should be presented appropriately and supported by the data.

2. Please review the manuscript carefully for clarity, consistency, and readability, including terminology, tables and figures, statistical reporting, and the alignment among the abstract, results, discussion, and conclusion.

[Note: HTML markup is below. Please do not edit.]

Reviewers' comments:

**Comments to the Author**

Reviewer #1: All comments have been addressed

Reviewer #2: All comments have been addressed

**********

2. Is the manuscript technically sound, and do the data support the conclusions?

Reviewer #1: Yes

Reviewer #2: Yes

**********

3. Has the statistical analysis been performed appropriately and rigorously?

Reviewer #1: Yes

Reviewer #2: Yes

**********

4. Have the authors made all data underlying the findings in their manuscript fully available?

The PLOS Data policy

Reviewer #1: Yes

Reviewer #2: Yes

**********

5. Is the manuscript presented in an intelligible fashion and written in standard English?

Reviewer #1: Yes

Reviewer #2: Yes

**********

Reviewer #1: Thank you for the authors’ careful revisions and hard work. I am pleased to note that my previous concerns have been fully addressed.

Reviewer #2: Thank you for the careful revision. The manuscript has improved substantially, and most of my previous concerns have been addressed. The revised title is more precise, the task classifications and source manipulation are explained more clearly, and the interpretation of the findings is generally more cautious.

1. Please provide the remaining statistical clarifications.

In response to my previous comment, the manuscript now clarifies that participant-level random intercepts were included. However, several requested details remain unclear. Please state whether continuous predictors were centered or standardized, explain how the simple slope analyses were conducted, and clarify whether random slopes were considered. Please also explain how potential edge cases in the conformity index were handled, particularly trials in which the denominator could equal zero.

2. Please further soften one interpretive claim.

The statement that the findings “indicate” that socially disconnected individuals may not readily adopt AI as a relational substitute remains somewhat stronger than the current data allow. Because relational substitution and social approach motivations were not directly measured, please present this as a possible interpretation rather than a conclusion directly established by the study.

3. Please correct the remaining language issues.

A final language check is needed. For example, “a significant main effects” should be revised to “a significant main effect,” and “the relationship between social exclusion and conformity depending on opinion source” should be revised to “the relationship between social exclusion and conformity depended on opinion source.” The corresponding sentence regarding loneliness should also be corrected.

4. Please clarify the funding statement.

The submission form states that the authors received no specific funding, whereas the Acknowledgements section states that the work was partially supported by the Department of Psychology at The Catholic University of Korea. Please ensure that these statements are consistent and accurately reported.

Thank you again for the careful revision.

**********

what does this mean?). If published, this will include your full peer review and any attached files.

If you choose “no”, your identity will remain anonymous but your review may still be made public.

**Do you want your identity to be public for this peer review?**  For information about this choice, including consent withdrawal, please see our Privacy Policy

Reviewer #1: No

Reviewer #2: No

**********

[NOTE: If reviewer comments were submitted as an attachment file, they will be attached to this email and accessible via the submission site. Please log into your account, locate the manuscript record, and check for the action link "View Attachments". If this link does not appear, there are no attachment files.]

To ensure your figures meet our technical requirements, please review our figure guidelines: https://journals.plos.org/plosone/s/figures

You may also use PLOS’s free figure tool, NAAS, to help you prepare publication quality figures: https://journals.plos.org/plosone/s/figures#loc-tools-for-figure-preparation.

NAAS will assess whether your figures meet our technical requirements by comparing each figure against our figure specifications.

# Author comment: Author response to Decision Letter 2

**DOI:** 10.1371/journal.pone.0355478.r004

6 Jul 2026

Reviewer 2.

Review 1

The reviewer requested additional statistical clarifications. Specifically, the reviewer asked us to clarify whether continuous predictors were centered or standardized, how the simple slope analyses were conducted, whether random slopes were considered, and how potential edge cases in the conformity index were handled, particularly cases in which the denominator could equal zero.

Response

Thank you for requesting these additional statistical clarifications. We have now clarified these points in the revised manuscript.

First, continuous predictors were entered into the models without mean-centering. Standardized regression coefficients (β) were reported to facilitate interpretation and comparison across predictors.

Second, we clarified how the simple slope analyses were conducted. To probe the significant interaction between social exclusion and opinion source, we estimated the simple effect of social exclusion on conformity separately within the AI and human opinion-source conditions. The same procedure was applied to the loneliness × opinion source interaction.

Third, regarding the random-effects structure, random slopes were not specified or estimated in the final models. The mixed-effects models included participant-level random intercepts to account for repeated observations nested within participants. Thus, the random-effects structure used in the analyses was a random-intercept-only structure.

Finally, we clarified the issue of potential edge cases in the conformity index. In the present study, the denominator of the conformity index could not equal zero by design. Specifically, the reference values used in the conformity calculation were decimal values, whereas participants’ responses were restricted to integer Likert-type values. Therefore, participants’ initial responses could not be identical to the reference value used in the denominator.

Review 2

The reviewer noted that the statement that the findings “indicate” socially disconnected individuals may not readily adopt AI as a relational substitute was stronger than warranted by the present data. Because relational substitution and social approach motivations were not directly measured, the reviewer asked us to present this point as a possible interpretation rather than as a conclusion directly established by the study.

Response

Thank you for this helpful suggestion. We agree that the previous wording was stronger than warranted because the present study did not directly measure relational substitution or social approach motivations. We have therefore softened this claim in the revised manuscript. Specifically, we revised the statement to frame it as a possible interpretation rather than as a conclusion directly established by the data. The revised text now states that one possible interpretation is that socially disconnected individuals may not necessarily adopt artificial agents as relational substitutes, while also noting that this interpretation remains tentative and should be examined more directly in future research.

Review 3

The reviewer requested a final language check and identified several remaining grammatical issues, including the phrases “a significant main effects,” “the relationship between social exclusion and conformity depending on opinion source,” and the corresponding sentence regarding loneliness.

Response

Thank you for identifying these remaining language issues. We have corrected “a significant main effects” to “a significant main effect.” We also revised the sentence concerning social exclusion to state that “the association between social exclusion and conformity differed by opinion source,” and corrected the corresponding sentence regarding loneliness in the same way. In addition, we conducted a final language check throughout the manuscript to improve clarity and readability.

Attachment Submitted filename: Response.docx

# Editor report: Decision Letter 2

**DOI:** 10.1371/journal.pone.0355478.r005

**Authors:** Lianshan Zhang

22 Jul 2026

Conforming to AI vs. humans: The moderating effects of social exclusion and task type

PONE-D-26-10716R2

Dear Dr. Jeong,

We’re pleased to inform you that your manuscript has been judged scientifically suitable for publication and will be formally accepted for publication once it meets all outstanding technical requirements.

Within one week, you’ll receive an e-mail detailing the required amendments. When these have been addressed, you’ll receive a formal acceptance letter and your manuscript will be scheduled for publication.

An invoice will be generated when your article is formally accepted. Please note, if your institution has a publishing partnership with PLOS and your article meets the relevant criteria, all or part of your publication costs will be covered. Please make sure your user information is up-to-date by logging into Editorial Manager at Editorial Manager® and clicking the ‘Update My Information' link at the top of the page. For questions related to billing, please contact billing support.

If your institution or institutions have a press office, please notify them about your upcoming paper to help maximize its impact. If they’ll be preparing press materials, please inform our press team as soon as possible -- no later than 48 hours after receiving the formal acceptance. Your manuscript will remain under strict press embargo until 2 pm Eastern Time on the date of publication. For more information, please contact onepress@plos.org.

Kind regards,

Lianshan Zhang

Academic Editor

PLOS One

Additional Editor Comments (optional):

Reviewers' comments:

# Editor report: Acceptance letter

**DOI:** 10.1371/journal.pone.0355478.r006

**Authors:** Lianshan Zhang

PONE-D-26-10716R2

PLOS One

Dear Dr. Jeong,

I'm pleased to inform you that your manuscript has been deemed suitable for publication in PLOS One. Congratulations! Your manuscript is now being handed over to our production team.

At this stage, our production department will prepare your paper for publication. This includes ensuring the following:

* All references, tables, and figures are properly cited

* All relevant supporting information is included in the manuscript submission,

* There are no issues that prevent the paper from being properly typeset

You will receive further instructions from the production team, including instructions on how to review your proof when it is ready. Please keep in mind that we are working through a large volume of accepted articles, so please give us a few days to review your paper and let you know the next and final steps.

Lastly, if your institution or institutions have a press office, please let them know about your upcoming paper now to help maximize its impact. If they'll be preparing press materials, please inform our press team within the next 48 hours. Your manuscript will remain under strict press embargo until 2 pm Eastern Time on the date of publication. For more information, please contact onepress@plos.org.

You will receive an invoice from PLOS for your publication fee after your manuscript has reached the completed accept phase. If you receive an email requesting payment before acceptance or for any other service, this may be a phishing scheme. Learn how to identify phishing emails and protect your accounts at https://explore.plos.org/phishing.

If we can help with anything else, please email us at customercare@plos.org.

Thank you for submitting your work to PLOS ONE and supporting open access.

Kind regards,

PLOS ONE Editorial Office Staff

on behalf of

Dr. Lianshan Zhang

Academic Editor

PLOS One
